# Engaging Community in Research: Accelerating Translation of Research Into Practice

**DOI:** 10.1093/geroni/igaf122.429

**Published:** 2025-12-31

**Authors:** Susan Stark

**Affiliations:** Washington University in St. Louis, St. Louis, Missouri, United States

## Abstract

Community engaged research is an approach that embraces partnerships between researchers and their communities that encourages research designed for dissemination and implementation. This session describes the continuum of community-engaged research used by the Stark Lab in St. Louis: community partnerships, participant advisory boards, and a community-based research network and how each of these has improved the quality, applicability, and translatability of research done in the Lab related to fall intervention, home modifications, and social participation. Benefits of these approaches for the Lab include higher participant recruitment rates for studies, better cultural competence of research team members, increased university-community trust, ability to tailor and adapt interventions to meet different community needs, intensive community-engaged skill-building for students and trainees. Benefits for community partners include improved community knowledge and capacity, greater readiness to adopt interventions (e.g. fall prevention programs), reduced gaps in service delivery, improved communication between community based organizations, and increased access to evidence and training. Challenges include time, funding, and availability for university and community-based partners. However, benefits far outweigh challenges and solutions such as building funding to support these efforts into grant funding is helpful.

